# Efficacy of wearing compression garments during post-exercise period after two repeated bouts of strenuous exercise: a randomized crossover design in healthy, active males

**DOI:** 10.1186/s40798-017-0092-1

**Published:** 2017-07-03

**Authors:** Kazushige Goto, Sahiro Mizuno, Ayaka Mori

**Affiliations:** 1 Faculty of Sport and Health Sciences, Ritsumeikan University, 1-1-1, Nojihigashi, Kusatsu, Shiga 525-8577 Japan; 2 Graduate School of Sport and Health Science, Ritsumeikan University, Kusatsu, Shiga 525-8574 Japan

**Keywords:** Post-exercise treatment, Fatigue, Exercise-induced muscle damage, Muscle function

## Abstract

**Background:**

The efficacy of wearing [a] compression garment (CG) between repeated bouts of exercise within a same day has not been fully understood. The present study determined the effect of wearing a CG after strenuous exercise sessions (consisting of sprint exercise, resistance exercise, drop jump) twice a day on exercise performance, muscle damage, and inflammatory responses.

**Methods:**

Eleven physically active males (age, 22.7 ± 0.9 years; height, 175.7 ± 6.7 cm; body mass, 73.6 ± 10.2 kg; BMI, 23.8 ± 2.7 kg/m^2^) performed two trials (a randomized crossover design), consisting of the trial with either wearing a whole-body CG during post-exercise period (CG trial) or the trial with wearing a normal garment without specific pressure (CON trial). Two exercise sessions were conducted in the morning (09:00–10:00, Ex1) and afternoon (14:00–15:00, Ex2). Immediately after completing 60 min of each exercise, the subjects in the CG trial changed into a whole-body CG. Time-course changes in exercise performance (bench press power, jump performances, repeated sprint ability), blood variables (lactate, glucose, myoglobin, creatine kinase, interleukin-6, leptin), and scores of subjective feeling (fatigue, muscle soreness) were compared between the CG and CON trials before Ex1 (8:40), immediately before Ex2 (14:00, 4 h after Ex1), 4 h after Ex2 (19:00), and 24 h after the onset of Ex1 (9:00).

**Results:**

Two bouts of exercise significantly decreased performances of counter movement jump (main effect for time: *P* = 0.04, *F* = 3.75, partial *η* ^2^ = 0.27) and rebound jump (main effect for time: *P* = 0.00, *F* = 12.22, partial *η* ^2^ = 0.55), while no significant difference was observed between the two trials (interaction: *P* = 0.10, *F* = 1.96, partial *η* ^2^ = 0.16 for counter movement jump, *P* = 0.93, *F* = 0.01, partial *η* ^2^ = 0.001 for rebound jump). Repeated sprint ability (power output during 10 × 6 s maximal sprint, 30-s rest periods between sprints) did not differ significantly between the two trials at any time points. Power output during bench press exercise was not significantly different between the two trials (interaction: *P* = 0.46, *F* = 0.99, partial *η* ^2^ = 0.09 for Ex1, *P* = 0.74, *F* = 0.38, partial *η* ^2^ = 0.04 for Ex2, *P* = 0.22, *F* = 1.54, partial *η* ^2^ = 0.13 for 24 h after the onset of Ex1). Serum myoglobin, creatine kinase, leptin, and plasma interleukin-6 were not significantly different between the two trials (interaction: *P* = 0.16, *F* = 2.23, partial *η* ^2^ = 0.18 for myoglobin; *P* = 0.39, *F* = 0.81, partial *η* ^2^ = 0.08 for creatine kinase; *P* = 0.28, *F* = 1.30, partial *η* ^2^ = 0.13 for leptin; *P* = 0.34, *F* = 1.05, partial *η* ^2^ = 0.12 for interleukin-6). Muscle soreness at 24 h during post-exercise period was significantly lower in the CG trial than in the CON trial for pectoralis major muscle (*P* = 0.04), while the value was inversely lower in the CON trial for hamstring (*P* = 0.047).

**Conclusions:**

Wearing a whole-body CG during the post-exercise period after two bouts of strenuous exercise sessions separated with 4 h of rest did not promote recovery of muscle function for lower limb muscles nor did it attenuate exercise-induced muscle damage in physically active males.

## Key points

- Wearing a whole-body compression garment during the post-exercise period did not markedly affect recovery of muscular strength.
- Indirect muscle damage markers in blood (e.g., serum myoglobin, creatine kinase) were not influenced by wearing a compression garment during the post-exercise period.
- It was likely that the use of compression garments during the post-exercise period may have had some favorable effect on recovery of power output for upper body muscles.

## Background

Since athletes commonly perform intensive physical training or competitions on consecutive days, facilitation of recovery process is important to maximize competitive success and to prevent excessive fatigue [1]. Several strategies are currently employed on sports fields to aid the recovery process, including massage [2], active recovery [3], water immersion [2], contrast bathing [4], and hyperbaric oxygen supply [5]. In addition, the use of compression garments (CG) during post-exercise period has been recently increasing attention as a novel option to promote muscular strength recovery and to attenuate exercise-induced muscle damage [6–8].

Although some evidences exist for beneficial effects of wearing a CG during exercise [9–12], majority of previous studies failed to support the performance-enhancing effect of the use of a CG during exercise [8, 13–19]. In a latest review [20], CG did not reveal positive effects on running performance, maximal and submaximal oxygen uptake, or the performance of strength-related tasks after running. In addition, MacRae et al. [8] suggested that the use of CG may have had some help for certain aspects of jump performance in some situations [9]. However, only limited evidence [12] showed the beneficial effects of CG on the performance of other exercise types (e.g., pedaling exercise, running). Alternatively, improvement of recovery by wearing a CG during post-exercise period is more apparent [6, 7, 10, 21–24]. Kraemer et al. [7] demonstrated that wearing a whole-body CG for 24 h after resistance training caused rapid recovery of power output for the bench press throw and attenuated muscle soreness with lower creatine kinase (CK) concentration on the following morning. Jakeman et al. [6, 22] reported that the recovery of jump performance following 100 plyometric drop jumps was significantly improved when the subjects wore a CG during post-exercise period. Furthermore, we have previously reported that recovery of muscular strength for upper limb muscles was significantly improved during early phase (3–8 h) of post-resistance exercise period by wearing whole-body CG. However, for the lower limb muscle, a significantly faster recovery of muscular strength occurred at 24 h after exercise [24]. In a previous study using endurance exercise, wearing CG for 24 h after 30 min of downhill running promoted significantly recovery of counter movement jump height [25]. Potential factor for promoted recovery by CG during post-exercise period is suggested to be reductions of venous blood pooling and subsequent swelling in muscles [26]. In addition, Born et al. [27] pointed out that the use of CG during post-exercise may assist performance recovery.

Athletes are often required to conduct strenuous exercise or competition twice a day, separated with several hours (12 h <) of rest. However, the influence of wearing CG between the repeated bouts of exercise within a same day has not been fully understood. Duffield et al. [26] reported that combined treatment of cold water immersion (15 min at 10 °C) and wearing CG (during 3 h) after the tennis specific drill and match play sessions promoted recovery of counter movement jump (CMJ) height at the beginning of subsequent match play. However, due to the recovery enhancing effect by cold water immersion [28–30], the impact of the use of CG itself has not been identified.

Therefore, the purpose of the present study was to determine effect of the CG during post-exercise period after two repeated bouts of exercise (including repeated sprint exercise, resistance exercise, drop jump) on exercise performance (e.g., power output during bench press exercise, jump height, rebound jump index), muscle damage, and inflammatory responses. We hypothesized that the use of CG during post-exercise period after the two repeated bouts of exercise would promote recovery of muscle function.

## Methods

### Subjects

Eleven men (mean ± SD: age, 22.7 ± 0.9 years; height, 175.7 ± 6.7 cm; body weight, 73.6 ± 10.2 kg; BMI, 23.8 ± 2.7 kg/m^2^) participated in the present study. None of them was taking part in any regular training program at the start of the experiment (with exercising recreationally once per week). However, all subjects had several years of experience performing strenuous resistance training. The inclusion criteria for subject selection were experience with strenuous resistance training at least a year, no habit of wearing CG in daily sport activities. The subjects were informed about the purpose of the study and the experimental procedures, and they provided a written informed consent. The present study was approved by the Ethics Committee for Human Experiments at Ritsumeikan University, Japan, in accordance with the Helsinki Declaration.

### Experimental overview

The present study was performed with a randomized crossover design. The subjects visited the laboratory four times throughout the experimental period. On the first visit, all subjects provided a written informed consent. On the second visit, one-repetition maximum (1RM) for four exercises was measured to determine the weights to be used for each exercise on the experimental days. The subjects also conducted a familiarization session of exercise protocol, consisting of 10 × 6 s all-out sprint separated with 30-s rest between the sprints under 7.5% of each body weight, 10 × 3 sets resistance exercise for four exercises, and 5 × 10 drop jumps (50 jumps in total).

**Fig. 1:**
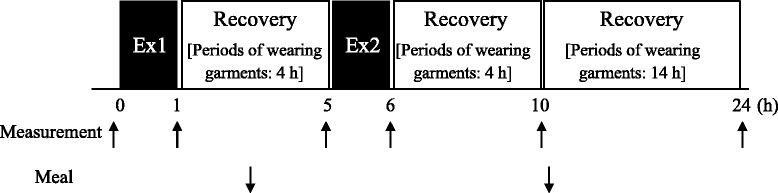
Timeline of measurements and meal consumption. All subjects performed two bouts of exercise sessions (Ex1, Ex2) with wearing normal garment, while they kept wearing either CG (CG trial) or normal garment (CON trial) during the recovery period

On the third and fourth visits, the subjects completed two experimental trials, either with the use of a CG (CG trial) or without the use of a CG (CON trial) during post-exercise period after performing two repeated bouts of exercise, separated with 4 h of rest (Ex1: 9:00–10:00, Ex2: 14:00–15:00). The CG and CON trials were conducted in a random order separated by a month. Immediately after completing 60 min of each exercise, the subjects in the CG trial changed into a whole-body CG (Recharge; Under Armour, Baltimore, MD) [7, 24]. The pressure levels applied for the present CG were previously reported [25], 11.5 ± 0.6 hPa for thigh and 17.6 ± 1.8 hPa for calf. In the CON trial, the subjects wore a non-CG, identical type of sports wear without specific compression. The appropriate size of the CG for each subject was chosen on the basis of the garment’s instruction manual and involved measurements of the height, chest, waist, and ankle circumferences. The subjects wore the prescribed garments throughout the whole recovery period (4 h after Ex1 and approximately 18 h after Ex2), except during two repeated bouts of exercise (60 min for each exercise), during measurements of exercise performances, blood drawing, and showering at night. Time courses of changes in upper and lower body muscular strength and power, blood metabolites, hormone and cytokine levels, and scores of muscle soreness and fatigue were monitored during 24 h after the onset of Ex1 (Fig. 1).

On the experimental days, all subjects stayed at the same facility located in the university. They spent time by reading books, listening music, or watching DVD. They were allowed to consume water ad libitum. The subjects were given identical lunch (12:00) and dinner (19:00) in both trials. The sleep duration on exercise days was controlled from 23:00 to 07:00.

### Strenuous exercise session

Exercise consisted of three different exercises to mimic regular training for improving fitness levels among team sport athletes, including repeated sprint exercise, resistance exercise, and drop jump. We selected this protocol because all exercises are commonly used for daily training on sport fields. For the repeated sprint exercise, the subjects completed 10 × 6 s all-out sprint separated with 30 s rest between the sprints using an electromagnetically braked cycle ergometer (Power Max VIII; Konami Corporation, Tokyo, Japan). The resistance of pedaling was set at 7.5% of each body weight. Resistance exercise consisted of four exercises: three exercises for upper body muscles (chest press, lat pull down, shoulder press) and an exercise for lower limb muscles (bilateral leg press) using weight stack machines (Life Fitness, Ltd., Tokyo, Japan). Each exercise involved 10 repetitions, with five sets for chest press and lat pull down and three sets for shoulder press and bilateral leg press. The resistance was set as 75% of 1RM for each exercise. The subjects rested for 2 min between sets and exercises. Before the training session in each trial, the subjects performed warm-up sets comprising 10 repetitions at 50% of the 1RM and stretching of the major muscle groups targeted by the exercises. For drop jump, the subjects completed 5 × 10 drop jumps (50 jumps in total) from a height of 40 cm. All jumps were performed with placing hands on hips. After landing, they are requested to pause at a squatting position, with hand on hips and knees flexed to approximately 90° and subsequently conducted vertical jump with maximal effort [6, 22]. Each exercise session including repeated sprint exercise, resistance exercise, and drop jump lasted 60 min. The exercise session was repeated twice in the morning (9:00–10:00, Ex1) and afternoon (14:00-15:00, Ex2) under supervision by laboratory staff.

### Measurements

Before Ex1 (8:40), immediately after Ex1 (10:00), immediately before Ex2 (14:00, 4 h after Ex1), 4 h after Ex2 (19:00), and 24 h after the onset of Ex1 (9:00), maximal power for bench press, jump performance, blood variables, scores of fatigue, and muscle soreness were evaluated. The repeated sprint ability was also evaluated three times: during Ex1, during Ex2, and at 24 h during post-exercise period (Fig. 1).

### Evaluation of repeated sprint ability

To evaluate repeated sprint ability, the subjects performed repeated sprint exercise, comprising 10 × 6 s all-out sprint with a 30-s rest period between sprints. Before the exercise, the subjects completed a standardized warm-up on an electromagnetically braked cycle ergometer (Power Max VIII; Konami Corporation, Tokyo, Japan). The applied load for the repeated sprint test was equivalent to 7.5% of the subjects’ body weight. The mean power outputs during each set of sprint were recorded by a computer (Edge E420, Lenovo, Beijing, China) every 0.1 s using specially designed software (Konami, Tokyo, Japan). The power output decrement (%) was calculated by percentage reduction of power output over the 10 sprints.

### Strength measurement

For the indication of muscular power output for upper and lower limb muscles, bench press power output and jump performances were evaluated. Power output for bench press power output during concentric phase (elevating phase) was determined using an accelerometer (Myotest SPORT, Myotest SA, Sion, Switzerland) connective to a bench press bar [25]. After the beep, the subjects completed bench press exercise during concentric (elevating) phase as fast as possible. The weight of the exercise was set equivalent to 40% of 1RM, and mean power output (MPO) during the elevating phase was calculated. The measurement was repeated three times, and the maximal value was adopted.

Jump performance was evaluated using two types of jump tests. For the CMJ test, the subjects performed a maximal vertical jump on a platform (CT-916, Takei Scientific Instruments Co. Ltd., Niigata, Japan) that was connected to a personal computer. Subjects were instructed to perform a maximal jump while placing hands on the lumbar division to eliminate upper limb effects. The vertical jump flight time was recorded. From the flight time, the CMJ height was calculated using the formula [(Jump height (m) = 1/8 (flight time)^2^ × (the gravity constant)]. The rebound jump (RJ) test was then performed to evaluate stretch shortening ability for lower limb muscles. The subjects were instructed five repeated maximal jumps on a platform with minimum contact time, and the jump height, contact time, and RJ index (jump height/contact time) were calculated [6]. From the obtained results from the five jumps, the average value among three values except the highest and lowest values was adopted for further analysis.

### Blood variables

Venous blood samples were obtained from an antecubital vein before Ex1, immediately after Ex1, immediately before Ex2 (4 h after Ex1), 4 h after Ex2, and 24 h after the onset of Ex1 to determine blood glucose and lactate concentrations. Serum creatine kinase (CK), myoglobin (Mb), leptin, and plasma interleukin-6 (IL-6) concentrations were also evaluated before Ex1 and at 24 h after the onset of Ex1. Serum and plasma samples were obtained by centrifuging for 10 min and were stored at −80 °C until analysis. Serum CK and Mb concentrations were measured at a clinical laboratory (SRL Inc., Tokyo, Japan). The intra-assay CVs were 3.4% for CK and 6.0% for Mb measurements. Serum leptin and plasma IL-6 concentrations were measured with enzyme-linked immunosorbent assay (ELISA) using kits from R&D Systems (Minneapolis, MN, USA). The intra-assay CV was 9.5% for leptin and 8.2% for IL-6, respectively.

The blood glucose and lactate concentrations were measured immediately after blood collection using an automatic glucose analyzer (Free Style, Nipro Corporation, Osaka, Japan) and lactate analyzer (Lactate Pro2; Arkray Inc. Kyoto, Japan), respectively.

### Scores of fatigue and muscle soreness

Scores of subjective fatigue and vitality were evaluated six times (before Ex1, immediately after Ex1, before Ex2, immediately after Ex2, 4 h after Ex2, 24 h after the onset of Ex1) using a 100-mm visual analogue scale (VAS), where 0 mm represented “no fatigue (or “filled vitality”) at all” and 100 mm represented “unbearable fatigue (or “no vitality at all”)” [21]. At 24 h after the onset of Ex1, muscle soreness was assessed using a 100-mm VAS, where 0 mm represented “no pain at all” and 100 mm represented “unbearable pain”. The subjects were asked to rate the feeling experienced by making the line.

### Statistical analysis

Data are expressed as means ± standard deviation (SD). For comparisons of time-course changes in exercise performance, blood variables, and subjective feeling of fatigue, a two-way analysis of variance (ANOVA) with repeated measures was initially applied. When the ANOVA revealed a significant interaction or main effect, a Tukey-Kramer test was performed for post hoc analyses. For comparison of scores of subjective feeling evaluated at 24 h after the onset of Ex1, a paired *t* test was applied. For all tests, *P* < 0.05 was considered significant.

## Results

**Fig. 2:**
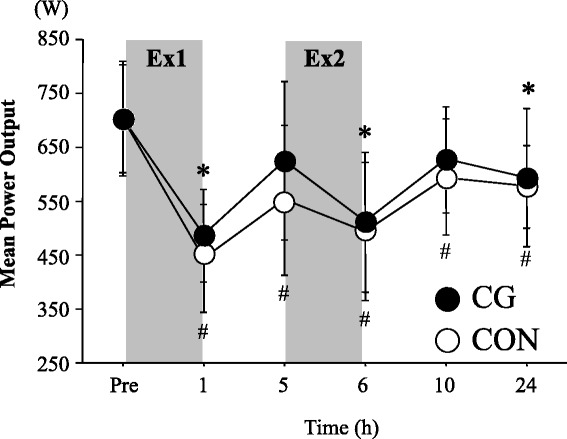
Mean power output for bench press exercise. *Shaded boxes* indicate duration of exercises. Values are means ± SD. *P < 0.05 vs. Pre (CG);^#^ *P* < 0.05 vs. Pre (CON)

Figure 2 presents time course of change in MPO during bench press. No significant interaction (*P* = 0.65, *F* = 0.67, partial *η* ^2^ = 0.06) or main effect for trial (*P* = 0.14, *F* = 2.63, partial *η* ^2^ = 0.21) was observed, and there was a significant main effect for time (*P* = 0.00, *F* = 24.60, partial *η* ^2^ = 0.71). In the CON trial, the MPO during bench press remained significantly lower from baseline value (Pre) at all time points during post-exercise period. However, in the CG trial, there was no significant difference from baseline value at 5 h (4 h after Ex1) and 10 h (4 h after Ex2) for the MPO. When the peak power output during the elevating phase of bench press exercise was compared, the CON trial showed significantly lower values from baseline value (Pre) at all points during post-exercise period (main effect for time: *P* = 0.00, *F* = 22.86, partial *η* ^2^ = 0.70). However, in the CG trial, there was no significant difference from the baseline value at 5 h (4 h after Ex1) and 10 h (4 h after Ex2), which were similar results from those for MPO.

**Fig. 3:**
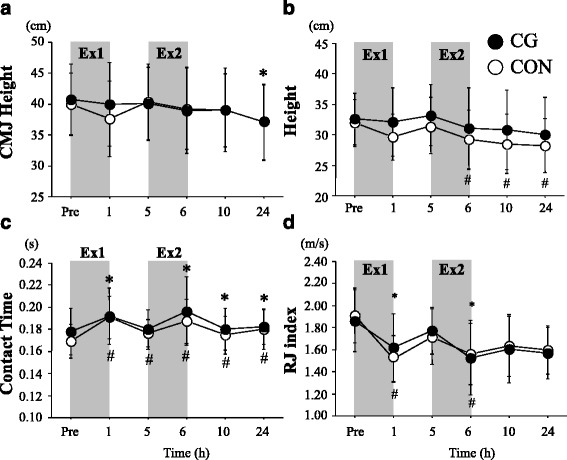
Results of CMJ (**a**) and RJ (**b**–**d**). Height of CMJ (**a**) and height (**b**), contact time (**c**), and index (**d**) during RJ are presented here. *Shaded boxes* indicate duration of exercises. Values are means ± SD. *P < 0.05 vs. Pre (CG); ^#^ *P* < 0.05 vs. Pre (CON)

Figure 3 presents time course of change in jump performance for CMJ and RJ. Exercise significantly reduced CMJ height during post-exercise period (main effect for time: *P* = 0.04, *F* = 3.75, partial *η* ^2^ = 0.27), with no significant interaction between trial and time (*P* = 0.10, *F* = 1.96, partial *η* ^2^ = 0.16). There was a significant main effect of time for all variables for RJ (RJ height: *P* = 0.02, *F* = 4.17, partial *η* ^2^ = 0.29, contract time: *P* = 0.00, *F* = 13.37, *η* ^2^ = 0.57, index: *P* = 0.00, *F* = 12.22, partial *η* ^2^ = 0.55). However, no significant interaction (RJ height: *P* = 0.66, *F* = 0.40, *η* ^2^ = 0.04, contract time: *P* = 0.42, *F* = 0.93, partial *η* ^2^ = 0.09, index: *P* = 0.17, *F* = 1.65, partial *η* ^2^ = 0.14) or main effect for trial (RJ height: *P* = 0.24, *F* = 1.58, partial *η* ^2^ = 0.14, contract time: *P* = 0.34, *F* = 0.99, partial *η* ^2^ = 0.09, index: *P* = 0.93, *F* = 0.01, partial *η* ^2^ = 0.001) was observed for any variables.

**Fig. 4:**
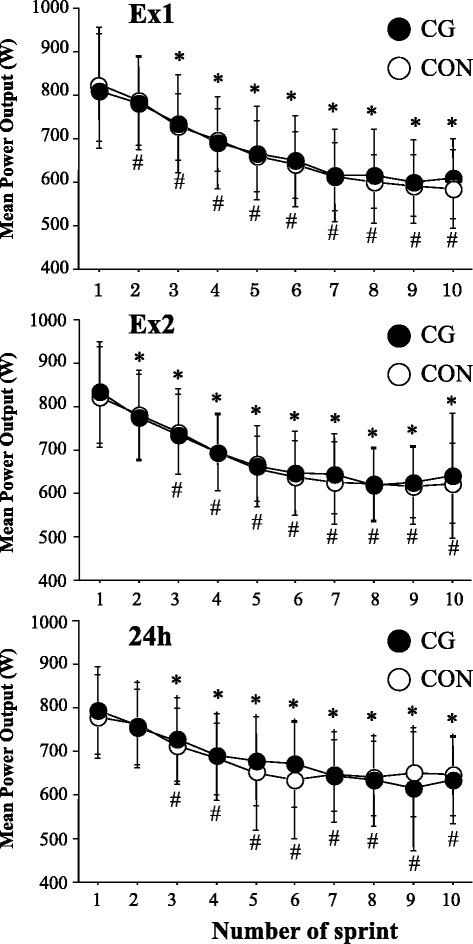
Power output during repeated sprint test. Values are means ± SD. *P < 0.05 vs. the value in the first sprint (CG); ^#^ *P* < 0.05 vs. the value in the first sprint (CON)

Figure 4 presents time course of changes in power output during repeated sprint test during Ex1 and Ex2 and at 24 h during the post-exercise period. Power output during repeated sprint test markedly decreased with progress of number of sprints (main effect for set: *P* = 0.00, *F* = 93.7, partial *η* ^2^ = 0.90 for Ex1, *P* = 0.00, *F* = 81.1, partial *η* ^2^ = 0.89 for Ex2, *P* = 0.00, *F* = 27.6, partial *η* ^2^ = 0.73 for 24 h). However, no significant interaction (trial × sprint) or main effect for trial was observed during Ex1 (interaction: *P* = 0.46, *F* = 0.99, partial *η* ^2^ = 0.09, main effect for trial: *P* = 0.72, *F* = 0.14, partial *η* ^2^ = 0.01), during Ex2 (interaction: *P* = 0.74, *F* = 0.38, partial *η* ^2^ = 0.04, main effect for trial: *P* = 0.42, *F* = 0.70, partial *η* ^2^ = 0.07), or at 24 h (interaction: *P* = 0.22, *F* = 1.54, partial *η* ^2^ = 0.13, main effect for trial: *P* = 0.65, *F* = 0.22, partial *η* ^2^ = 0.73) during the post-exercise period. Furthermore, power output decrement did not differ significantly between the two trials during Ex1, during Ex2, or at 24 h during post-exercise period (*P* > 0.05).

Blood lactate concentrations were markedly increased after Ex1 and Ex2 (main effect for time: *P* = 0.00, *F* = 73.5, partial *η* ^2^ = 0.88). However, there responses were similar between the CG and CON trials, and no significant difference between the trials was not observed at any time points (interaction: *P* = 0.38, *F* = 0.93, partial *η* ^2^ = 0.09). Similarly, exercise increased significantly blood glucose concentration (main effect for time: *P* = 0.00, *F* = 19.2, partial *η* ^2^ = 0.66), with no significant difference between the two trials (interaction: *P* = 0.18, *F* = 1.61, partial *η* ^2^ = 0.14, main effect for trial: *P* = 0.40, *F* = 0.76, partial *η* ^2^ = 0.07).

Table 1 presents changes in blood variables before exercise and at 24 h of post-exercise period. Before the exercise, there was no significant difference for any blood variables between the two trials. Serum Mb concentration did not change significantly after exercise (*P* = 0.16, *F* = 2.23, partial *η* ^2^ = 0.18), with no significant difference between the trials (interaction: *P* = 0.32, *F* = 1.09, partial *η* ^2^ = 0.10). Serum CK concentration significantly increased at 24 h during post-exercise period (main effect for time: *P* = 0.046, *F* = 5.17, partial *η* ^2^ = 0.34), and no significant interaction (trial × time) or main effect for trial was observed (interaction: *P* = 0.39, *F* = 0.81, partial *η* ^2^ = 0.08, main effect for trial: *P* = 0.26, *F* = 1.45, partial *η* ^2^ = 0.13). Plasma IL-6 and serum leptin concentrations did not change significantly at 24 h during post-exercise period (interaction: *P* = 0.34, *F* = 1.05, partial *η* ^2^ = 0.12 for plasma IL-6, *P* = 0.28, *F* = 1.30, partial *η* ^2^ = 0.13 for serum leptin).

**Table 1:** Changes in blood variables

			Pre			24 h	
Myoglobin (ng/mL)	CG	35	±	9	64	±	28*
	CON	32	±	6	56	±	36*
Creatine kinase (U/L)	CG	161	±	114	602	±	427*
	CON	110	±	34	544	±	286*
IL-6 (pg/mL)	CG	0.16	±	0.18	0.14	±	0.12
	CON	0.10	±	0.05	0.11	±	0.06
Leptin (ng/mL)	CG	1.4	±	1.7	1.3	±	1.8
	CON	1.5	±	1.8	1.1	±	1.5

Values are means ± SD

*Significantly different from Pre (*P* < 0.05)

The score of subjective muscle soreness at 24 h during post-exercise period was significantly lower in the CG trial than in the CON trial for pectoral major muscle [CG: 33 ± 21 mm, CON: 48 ± 25 mm, *P* = 0.04, d = 0.65], while the value was inversely lower in the CON trial than in the CG trial for hamstring [CG: 43 ± 24 mm, CON: 34 ± 26 mm, *P* = 0.047, d = 0.36]. There was no significant difference in scores of subjective muscle soreness for biceps branch, triceps brachii, or quadriceps femoris. Exercise significantly increased score of subjective fatigue (main effect for time: *P* = 0.00, *F* = 64.26, partial *η* ^2^ = 0.87). However, time course of change in subjective fatigue was not significantly different between the two trials during post-exercise period (interaction: *P* = 0.52, *F* = 0.85, partial *η* ^2^ = 0.08, main effect for trial: *P* = 0.75, *F* = 0.11, partial *η* ^2^ = 0.01).

## Discussion

In the present study, we have determined influence of wearing CG during post-exercise period on changes in exercise performance and exercise-induced muscle damage markers in response to two repeated bouts of training sessions separated with 4 h of rest period. Consequently, time-course changes in exercise performances for lower limb muscles or muscle damage markers in blood were similar between CG and CON trials. For the upper body muscles, no significant interaction (trial × time) or main effect for trial was found for MPO during bench press exercise. However, the use of CG revealed faster recovery of the MPO 4 h after the first bout (Ex1) and second bout (Ex2) of exercise sessions (*P* > 0.05 vs. baseline value), whereas in the CON trial, the MPO remained significantly lower throughout 24 h of post-exercise period.

The height of CMJ and performance variables for RJ did not differ significantly between CG and CON trials over 24 h of post-exercise period. Moreover, no significant difference in repeated sprint ability was observed between the two trials at any time points. These results differ from the findings from earlier studies in which wearing CG during post-exercise period promoted recovery of CMJ height [6, 22], MVC [6, 24], maximal isokinetic strength for lower limb muscles [22], and maximal power output during 5 min of pedaling [10]. In the present study, all subjects started wearing the CG from immediately after completing first bout of exercise (Ex1), and we have tested whether the recovery of muscle function was improved even during the early phase (4 h) of post-exercise period. In a previous study by Jakemen et al. [6], the subjects wore the CG for 12 h after 100 drop jumps. Consequently, recovery of performances for squat jump and CMJ was significantly improved by wearing CG at 24 h, but not at 1 h during post-exercise period. We have previously observed that wearing CG during post-exercise period facilitated significantly recovery of MVC for lower limb muscles at 24 h after resistance exercise. However, improved recovery of MVC was not observed at 1, 3, 5, and 8 h after the exercise [24]. Therefore, 4 h of wearing CG after the first exercise session may be insufficient to assist recovery of muscle function for lower limb muscles. Moreover, repeated sprint ability did not differ significantly between the two trials during Ex2 (at 4 h after completing Ex1) or at 24 h during post-exercise period. Because exercise-induced muscle damage impairs repeated sprint ability and sprint running performance [31], the use of CG during post-exercise period was expected to attenuate impairment of repeated sprint ability. The absence of improved repeated spring ability in the CG trial was inconsistent with a report [23] that showed increased performance for repeated sprint performance (10 × 40 m run) by wearing CG during 24 h of post-exercise period in rugby players. However, in the present study, repeated sprint ability during Ex2 and at 24 h during post-exercise period did not differ significantly from the value during Ex1 (baseline value) in either trial, suggesting that the exercise-induced decrement of power output was not evident during post-exercise period.

The MPO during bench press exercise significantly decreased immediately after Ex1 and Ex2 in both trials. However, in the CG trial, the MPO was recovered to baseline value following wearing CG for 4 h after both Ex1 and Ex2, while the values at the same time points remained significantly lower from baseline value in the CON trial. Influence of wearing CG on recovery of muscle function for upper body muscles has not been fully elucidated, but bench press throw power was significantly higher at 24 h after resistance exercise when the subjects wore the whole-body CG during post-exercise period. In contrast, promoted recovery of muscle function was not observed for lower limb muscles [7]. We have also shown that recovery of 1RM for chest press was significantly improved at 3, 5, and 8 h after the resistance exercise by wearing whole-body CG during post-exercise period [24]. Although somewhat inconsistent results exist [32], it is likely that wearing whole-body CG elicits recovery for upper body muscle function rather than for lower body muscles. However, in the present study, the recovery enhancing effect for upper body muscle function by wearing CG was smaller compared with two previous studies using resistance exercise protocols [7, 24]. The different outcome may be explained by difference in number of resistance exercise employed (four to six exercises in the previous studies vs. three exercises in the present study).

According to a recent systematic review and meta-analysis by Hill et al. [33], the use of CG after damaging exercise had a moderate effect in reducing the severity of muscle soreness and CK elevation and promoting recovery of muscle strength and power. In fact, 66% of the subjects analyzed (205 subjects in total from different studies) experienced reduced elevation of CK concentration. Similarly, Kraemer et al. [7] revealed that serum CK concentration at 24 h after resistance exercise was significantly lower after wearing whole-body CG during post-exercise period than the value after wearing non-compression garment. The score of muscle soreness for pectoral major muscle was significantly lower in the CG trial at 24 h during post-exercise period. Although mechanism for reduced muscle damage markers by wearing CG is still speculative, applied pressure by the garments generates an external pressure gradient that attenuates changes in osmotic pressure and reduces the space available for swelling and hematoma to occur [23]. A reduction of osmotic pressure with attenuating swelling may provide impaired inflammatory action and experience of soreness. In contrast, there were no significant differences between the two trials at 24 h during post-exercise period for serum CK, Mb, leptin, and plasma IL-6 concentrations. This result is not surprising, because the finding corresponds to previous reports presenting no influence of CG during post-exercise period on muscle damage markers in blood (e.g., CK, Mb, IL-6, C-reactive protein) [24, 34, 35].

Some limitations in the present study need to be considered carefully. In the present study, a psychological effect cannot be excluded because it is difficult to use CG in completely blinded conditions. However, we did not display detailed information of the prescribed CG, including expected outcomes and hypothesis. Furthermore, blood variables in the present study reflect physiological responses for both upper and lower body muscles, and we cannot clarify the differences in muscle damage and inflammatory responses between upper body and lower body muscles. Finally, we were not able to measure the pressure levels applied for the present subjects, although we have previously determined the pressure levels of the same CG among different subjects [25]. Therefore, it is possible that inter-individual differences of pressure levels and/or insufficient levels of pressure may have masked efficacy of the CG.

From practical viewpoints, the present findings may provide information regarding post-exercise treatment to promote recovery of maximal power output during training schedule with strenuous training sessions twice a day. The facilitation of recovery of muscular power output will be important to improve quality of subsequent training session, and wearing the CG during post-exercise period may have had some positive effects on recovery. Further researches are required to determine the efficacy of combined effects of CG and other traditional treatments (e.g., cold water immersion) on recovery of exercise performance in competitive athletes.

## Conclusions

In conclusion, wearing whole-body CG during post-exercise period after two bouts of exercise sessions separated with 4 h of rest period did not promote recovery of muscle function for lower limb muscles or did not affect exercise-induced muscle damage markers in blood among physically active males. However, it was likely that the use of CG during post-exercise period may have had some favorable effect on recovery of power output and severity of muscle soreness for upper body muscles.

## Abbreviations

1RM: One-repetition maximum
ANOVA: Analysis of variance
CG trial: Trial with wearing compression garment during post-exercise period
CK: Creatine kinase
CMJ: Counter movement jump
CON trial: Trial without wearing compression garment during post-exercise period
ELISA: Enzyme-linked immunosorbent assay
IL-6: Interleukin-6
Mb: Myoglobin
MPO: Mean power output
RJ: Rebound jump
SD: Standard deviation
VAS: Visual analogue scale
